# Heart Transplantation in a 14-Year-Old Boy in the Presence of Severe Out-of-Proportion Pulmonary Hypertension due to Restrictive Left Heart Disease: A Case Report

**DOI:** 10.1155/2013/418565

**Published:** 2013-04-11

**Authors:** Martin Schwienbacher, Ulrich Schweigmann, Nikolaus Neu, Elisabeth Schermer, Corinna Velik-Salchner, Ina Michel-Behnke, Erentraud Irnberger, Christina Maria Steger, Jörg Ingolf Stein, Ralf Geiger

**Affiliations:** ^1^Department of Pediatrics, Division of Cardiology, Pulmology, Allergology and Cystic Fibrosis, Innsbruck Medical University, 6020 Innsbruck, Austria; ^2^Department of Pediatrics, Pediatric Intensive Care Unit, Innsbruck Medical University, 6020 Innsbruck, Austria; ^3^Department of Anaesthesiology and Critical Care Medicine, Innsbruck Medical University, 6020 Innsbruck, Austria; ^4^Department of Pediatric Cardiology, Medical University of Vienna, 1090 Vienna, Austria; ^5^Department of Pediatrics, Salzburg State Hospital, 5020 Salzburg, Austria; ^6^Department of Pathology, Innsbruck Medical University, 6020 Innsbruck, Austria

## Abstract

A 14-year-old boy after balloon valvuloplasty of severe aortic valve stenosis in the neonatal period was referred for heart-lung transplantation because of high grade pulmonary hypertension and left heart dysfunction due to endocardial fibroelastosis with severe mitral insufficiency. After heart catheterization, hemodynamic parameters were invasively monitored: a course of levosimendan and initiation of diuretics led to a decrease of pulmonary capillary wedge pressure (from maximum 35 to 24 mmHg). Instead of an expected decrease, mean pulmonary artery pressures (mPAP) increased up to 80 mmHg with increasing transpulmonary pressure gradient (TPG) up to 55 mmHg. Oral bosentan and intravenous epoprostenol then led to a ~50% decrease of mPAP (TPG between 16 and 22 mmHg). The boy was listed solely for heart transplantation which was successfully accomplished 1 month later.

## 1. Case 

**Figure 1:**
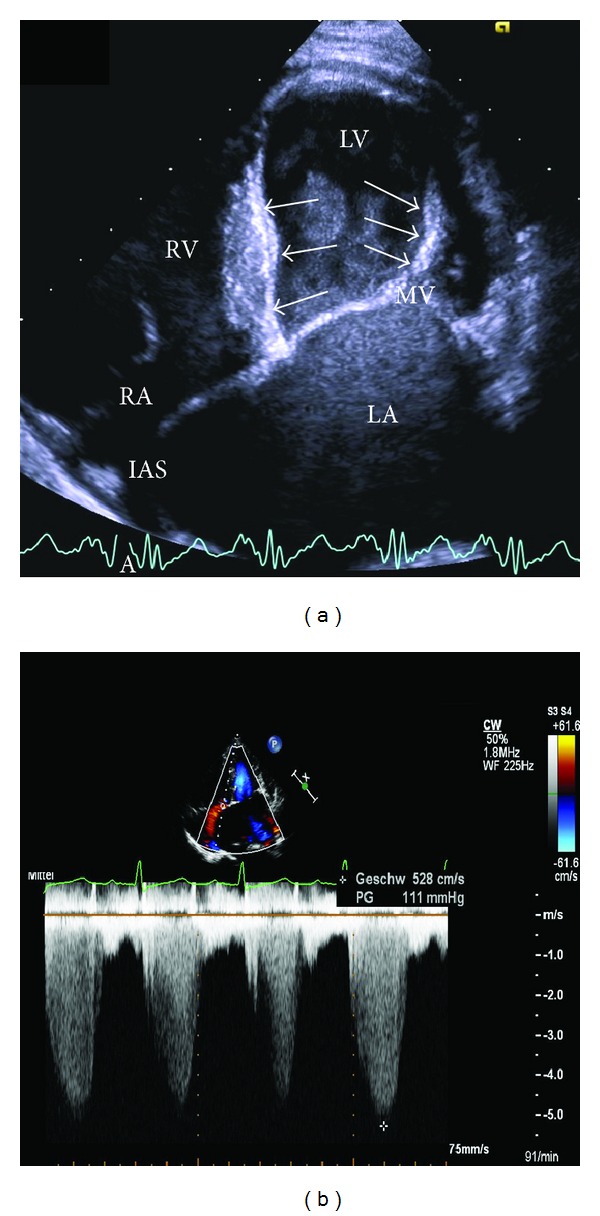
Echocardiography: 4-chamber view showing extremely enlarged left atrium (LA) and endocardial fibroelastosis (arrows) of left ventricle (LV) and mitral valve apparatus (a). Doppler maximum velocity of tricuspid insufficiency before initiation of medical therapy, indicating high systolic RV pressure of 111 mmHg (b). RV = right ventricle; RA = right atrium; IAS = interatrial septum.

A 14-year-old boy presented with the history of severe aortic valve stenosis; he had undergone successful balloon valvuloplasty at the age of 3 days. He was on followup elsewhere and was reportedly in stable clinical condition for 13 years, performing alpine skiing, cross country running, and mountain biking. He reportedly had normal systolic left heart function in the presence of left ventricular endocardial fibroelastosis (EFE) without signs of elevated right ventricular pressure on echocardiography. Neither heart catheterization nor cardiopulmonary exercise testing was performed. At the age of 14 years, he complained of fatigue during cycling and hiking for the first time. On echocardiography at that time severe mitral valve regurgitation and a markedly enlarged left atrium were seen besides the well-known mild aortic valve stenosis/regurgitation and endocardial fibroelastosis. Tricuspid valve regurgitation was demonstrated with a maximum velocity of 5.28 m/sec presuming a systolic pulmonary artery pressure of 111 mmHg (Figure 1). 

The boy was scheduled for heart catheterization. On admission, he was physically still active but had stopped sports. On physical examination, he was eupneic with a heart rate of 79 beats per minute at rest, blood pressure was 100/60 mmHg, and transcutaneous oxygen saturation (SaO_2_) was 97%. He had a 3/6 systolic heart murmur with punctum maximum over the third intercostal space at the left and the right parasternal border and a pronounced 2nd heart sound. 

**Figure 2:**
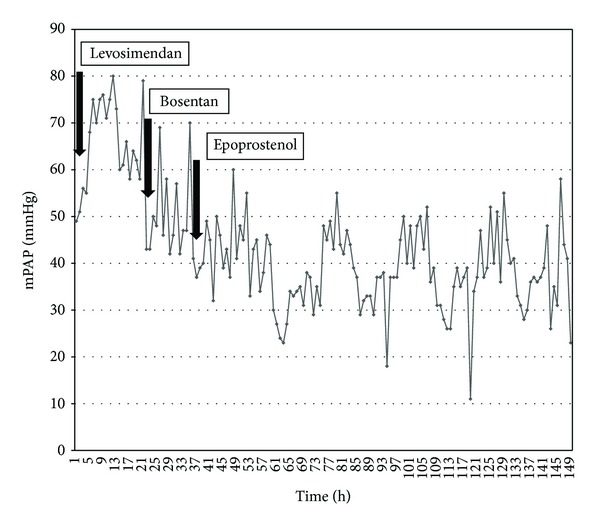
Original recording of invasively measured mean pulmonary artery pressures on ICU (days 1–5) and initiation of administration of levosimendan, bosentan, and epoprostenol.

Invasive measurements and calculations at baseline in conscious sedation at room air during heart catheterization were mean pulmonary artery pressure (mPAP) 58 mmHg, pulmonary capillary wedge pressure (PCWP) 30 mmHg, transpulmonary pressure gradient (TPG) 28 mmHg, cardiac index (CI) 2.6 l/min/m^2^, and pulmonary vascular resistance index (PVRI) 11 WU × m^2^; there was no pressure gradient across the aortic valve. On pulmonary vasoreactivity testing (100% oxygen + nitric oxide 20 ppm), mPAP increased to 67 mmHg, PCWP increased to 35 mmHg, TPG increased to 31 mmHg, and CI increased to 3.1 l/min/m^2^, while PVRI basically remained unchanged with 10 WU × m^2^. A Swan Ganz catheter was inserted, the patient was put on diuretics (furosemide 1 mg/kg/d, spironolactone 1 mg/kg/d), and a course of levosimendan (0.1 *µ*g/kg/min for 24 hours) was administered. After levosimendan, PCWP constantly decreased to values around 25 mmHg but mPAP increased up to 80 mmHg while cardiac index remained stable (Figure 2). 

**Figure 3:**
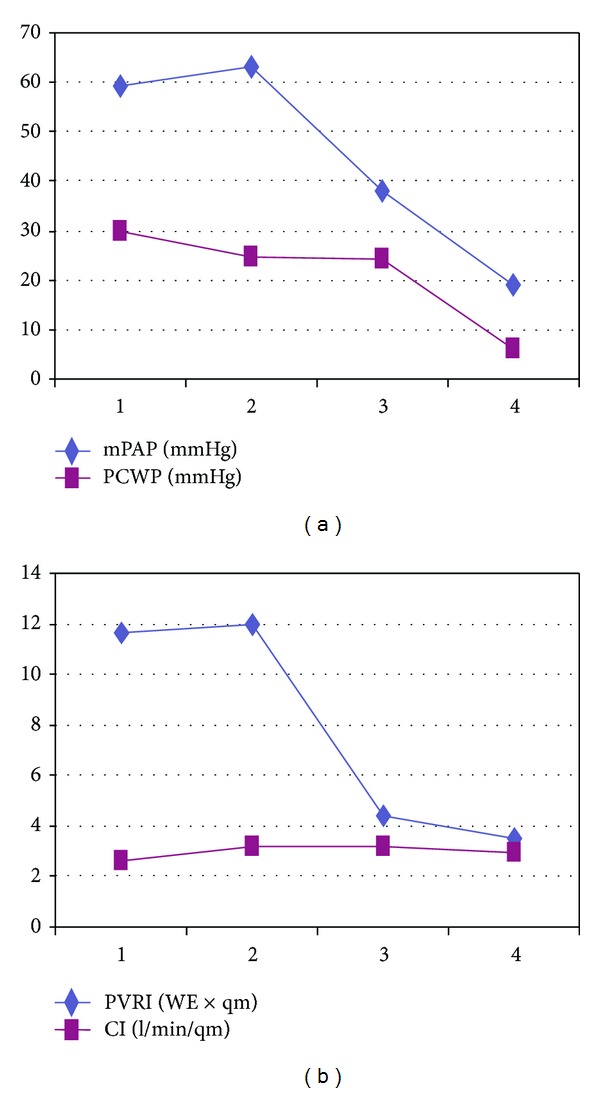
Hemodynamic measurements before and after heart transplantation. Mean pulmonary artery pressure (mPAP) pulmonary capillary wedge pressure (PCWP) (a). Pulmonary vascular resistance index (PVRI) and cardiac index (CI) (b). Time course before (1) and after levosimendan (2), with bosentan/epoprostenol (3) and after heart transplantation (4).

The markedly elevated TPG of up to 55 mmHg was taken for out-of-proportion pulmonary hypertension inadequate to the postcapillary component. Therefore, oral bosentan 125 mg/d and i.v. epoprostenol 2 ng/kg/min were started. This led to a substantial decrease of pulmonary vascular constriction, demonstrated by a decrease of mPAP to 38 ± 8 mmHG, without relevant changes in CI (values around 3.0 l/min/m^2^) and PCWP (values of around 24 mmHG). TPG thus decreased to values of 16 to 22 mmHg. Calculated PVRI was between 4 and 5 WU × m^2^ (Table 1, Figure 3).

The Swan Ganz catheter was removed 6 days later, and the patient was listed for heart transplantation. He remained stable on bosentan, epoprostenol, and diuretics until a donor heart became available. Heart transplantation was then successfully performed after 28 days on the waiting list. Perioperatively the patient received NO 20 ppm. Bosentan and epoprostenol were stopped on the first postoperative day and oral sildenafil 3 × 10 mg/d was initiated. The patient was weaned from NO two days after transplantation. His perioperative course was completely uneventful with a cardiac index of 2.5–3.5 l/min/m^2^, a PCWP of 12 mmHG, and mPAP of 30 mmHg immediately after transplantation. Heart catheterization three weeks later revealed normal PCWP (6 mmHg), mPAP (19 mmHg), PVRI (3.5 WU × m^2^), and TPG (13 mmHg) (Table 1). Four weeks after heart transplantation, sildenafil was stopped and the patient was discharged home.

**Figure 4:**
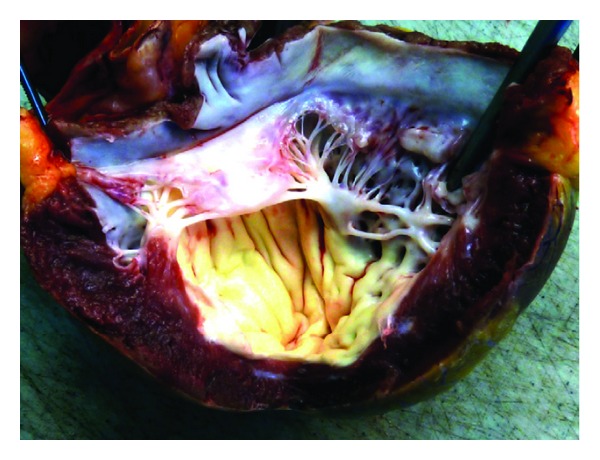
Pathologic specimen of the explanted heart. Endocardial fibroelastosis of the left ventricle affecting the papillary muscles with thickened and shortened chordae tendineae and the aortic and mitral valve (reprinted from [1] with permission from Elsevier).

Gross examination of the explanted heart showed globular enlargement and an extensive endocardial fibroelastosis of the left ventricle with involvement of the aortic and mitral valves, the papillary muscles, and chordae tendineae (Figure 4).

## 2. Discussion

Pulmonary hypertension (PH), secondary to structural or functional left heart disease in children, represents a delicate clinical condition where treatment options are often limited. The presence of endocardial fibroelastosis (Figure 4) in congenital aortic valve stenosis carries a poor prognosis; it is not correlated with the amount of aortic valve stenosis before or after intervention [2]. Reich et al. describe an incidence of 13.8% of EFE in long-term results of 269 patients after percutaneous balloon valvuloplasty [3]. It is thought that this fibroelastosis is a consequence of persistent pressure overload or decreased flow in the ventricle being present in early infancy and even in the fetus. Decreased diastolic function with preserved systolic function is present; therefore, diagnosis by means of echocardiography often is difficult. Left ventricular diastolic dysfunction leads to high filling pressures and enlargement of the left atrium. Pulmonary capillary wedge pressure rises, which leads to an increase in mPAP. This form of pulmonary hypertension is classified as pulmonary hypertension owing to left heart disease (group 2, Dana Point Classification) [4] characterized by an increased PCWP and a diastolic pulmonary artery pressure gradient of ≥10 mmHg. The TPG is calculated as the difference between the mean PA pressure and PCWP. By definition, it is flow-independent and thus may better reflect resistance to flow across the pulmonary bed. It is assumed that a TPG of around 15 mmHg is acceptable for heart transplantation, although there is no accurate definition yet. We and others consider pulmonary hypertension to be inadequately high to left heart disease when the mPAP is severely elevated in the presence of moderately elevated PCWP or LVEDP (≥22 mmHg) and a TPG of 18–20 mmHg [5]. These patients have a poor outcome after heart transplantation alone [6]; thus, a combined heart-lung transplantation is considered to be the only surgical option.

Medical treatment of pulmonary hypertension by advanced therapies without treating the underlying left heart disease can lead to severe pulmonary congestion [7]. Therefore, it is mandatory to test vasoreactivity of the pulmonary artery bed prior to therapy. The reversibility of increased PVR or TPG under pharmacologic testing is supposed to indicate a decreased probability of right ventricular failure/death after transplantation [8]. It has been shown that, for example, in patients with mitral stenosis, surgical or interventional decompression of the left atrium with a concomitant rapid decrease in the LA and PCWP is followed by a marked decrease in PA pressure. Lower TPG eventually resulted in normalization of the pulmonary artery pressure [9].

What is remarkable in this case is the substantial and somewhat unexpected increase of pulmonary artery pressure after improvement of left ventricular restrictive kinetics by diuretics and levosimendan. At that point, we felt safe enough to initiate pulmonary vasodilative therapy in order to lower PVR.

The positive inotropic action of levosimendan is due to its binding to troponin C which facilitates the interaction between actin and myosin filaments without changes in intracellular Ca++ ion concentrations [10, 11]. Because of its calcium sensitizing mechanism levosimendan leads even to a shorter isovolumic relaxation time [12]. In addition, the drug produces peripheral, coronary [13], and pulmonary vasodilation by opening ATP-sensitive potassium channels [14]. These mechanisms lead to an improved myocardial contractility and a better diastolic function of both the left and the right heart without increasing myocardial oxygen consumption. Improved right heart function and a previously “hidden” precapillary pulmonary vasoconstriction might have increased PAP and PVRI. A decrease in PAP and PVRI [15] and in filling pressures of the right ventricle [16], like in this case by vasodilating agents, could result in an increased contractility and mechanical effectiveness of the right ventricle [17, 18].

After consultation of several centers with superb expertise, surgical options like resection of EFE and mitral valve plasty as well as extra corporal mechanical support by means of left ventricular assist device were omitted in our patient and heart transplantation was considered as the therapeutic strategy with most favorable prognostic outcome for this young boy. 

Diuretics and levosimendan led to a substantial improvement of diastolic left heart function. Our own experience of beneficial effects of pulmonary vascular dilative medical therapy with bosentan in patients with heart failure with a normal ejection fraction with PVH and right ventricular failure [19] led us to consider the application of an endothelin-1-receptor antagonist in this case. In addition a therapy with epoprostenol, of which beneficial effects on reversibility of pulmonary hypertension in congestive heart failure prior to cardiac transplantation are known, was started [8, 20–22]. 

The pursued stepwise increase of epoprostenol from the initial dose of 2 ng/kg/min could not be carried out due to systemic hypotension (RR < 100/60 mmHg), most probably because of synergistic vasodilative effects of both drugs on the systemic vasculature. Therefore we consider bosentan as the main acting drug on the pulmonary vascular bed in this case. 

## 3. Conclusion 

Invasive monitoring of therapeutic effects on left heart function and pulmonary vasoreactivity is mandatory before deciding on heart or heart lung transplantation in patients with pulmonary hypertension owing to left heart disease.

## Figures and Tables

**Table 1:** Hemodynamic measurements and calculated values before and after heart transplantation: time course before (1) and after levosimendan (2), with bosentan/epoprostenol (3) and after heart transplantation (4).

	1	2	3	4	Units
sPAP	78	84	56	29	mmHg
mPAP	59	63	38	19	mmHg
dPAP	50	50	28	11	mmHg
PVRI	11.6	12	4.4	3.5	WU × m^2^
PCWP	30	25	24	6	mmHg
TPG	29	39	14	13	mmHg
sSAP	89	99	94	73	mmHg
mSAP	65	72	67	59	mmHg
dSAP	73	60	53	50	mmHg
SVRI	21.8	16.8	19.7	17.9	WU × m^2^
CI	2.6	3.2	3.2	3.7	L/min/m^2^

Systolic, mean, and diastolic pulmonary artery pressure (sPAP, mPAP, and dPAP); pulmonary vascular resistance index (PVRI); pulmonary capillary wedge pressure (PCWP); transpulmonary pressure gradient (TPG); systolic, mean, and diastolic systemic artery pressure (sSAP, mSAP, and dSAP); systemic vascular resistance index (SVRI); cardiac index (CI).
